# Retraction Note: Mechanical performance of sustainable high strength ductile fiber reinforced concrete (HSDFRC) with wooden ash

**DOI:** 10.1038/s41598-023-39437-3

**Published:** 2023-08-01

**Authors:** Jawad Ahmad, Rebeca Martínez-García, Jesús de-Prado-Gil, Amjad Ali Pasha, Kashif Irshad, Mostefa Bourchak

**Affiliations:** 1 Department of Civil Engineering, Swedish College of Engineering and Technology, Wah Cantt, Rawalpindi, Pakistan; 2 Department of Mining Technology, Topography, and Structures, University of León, Campus de Vegazana s/n, 24071 León, Spain; 3 Aerospace Engineering Department, King Abdulaziz University, 21589 Jeddah, Saudi Arabia; 4 Interdisciplinary Research Center for Renewable Energy and Power Systems, King Fahd University of Petroleum and Minerals, 31261 Dhahran, Saudi Arabia; 5 K.A.CARE Energy Research & Innovation Center at Dhahran, Dhahran, Saudi Arabia

Retraction of: *Scientific Reports* 10.1038/s41598-022-08134-y, published online 12 March 2022

The Editors have retracted this article.

After publication concerns were raised that the XRD spectra in Figure 13 are identical. The authors are unable to provide the original data for examination. In addition, an investigation by the Editors has shown inappropriate changes in authorship during the review process. The Editors no longer have confidence in the results and conclusions presented.

Jawad Ahmad, Amjad Ali Pasha and Kashif Irshad disagree with this retraction. Amjad Ali Pasha has stated on behalf of Mostefa Bourchak that they disagree with this retraction. The Editors were not able to obtain a current email address for Rebeca Martinez-Garcia and Jesús de-Prado-Gil.

